# Impact of Merit-Based Immigration Policies on Brain Drain From Low- and Middle-Income Countries

**DOI:** 10.1200/JGO.19.00266

**Published:** 2020-02-05

**Authors:** Nagi S. El Saghir, Benjamin O. Anderson, Julie Gralow, Gilberto Lopes, Lawrence N. Shulman, Hiba A. Moukadem, Peter Paul Yu, Gabriel Hortobagyi

**Affiliations:** ^1^ Division of Hematology Oncology, Department of Internal Medicine, American University of Beirut Medical Center, Beirut, Lebanon; ^2^ Fred Hutchinson Cancer Research Center, University of Washington, Seattle, WA; ^3^ Sylvester Comprehensive Cancer Center at the University of Miami and the Miller School of Medicine, Miami, FL; ^4^ Abramson Cancer Center, University of Pennsylvania, Philadelphia, PA; ^5^ Hartford HealthCare Cancer Institute, Hartford, CT; ^6^ The University of Texas MD Anderson Cancer Center, Houston, TX

## Abstract

**PURPOSE:**

Brain drain is the migration of educated and skilled individuals from a less developed region or country to a more economically established one. The Trump administration proposed a merit-based immigration plan. This article addresses its potential impact on health care delivery in low- and middle-income countries (LMICs) and their preparedness to deal with it.

**MATERIALS AND METHODS:**

Data on immigration policies, numbers of international medical graduates practicing in high-income countries (HICs), various scientific exchange methods, and efforts for capacity building in LMICs.

**RESULTS:**

Talented individuals seek to advance their knowledge and skills, and may stay in HICs because of greater rewards and opportunities. HICs also rely on immigrant international medical graduates to supplement their physician workforces.

**CONCLUSION:**

Ambitious individuals from LMICs need and should have opportunities to advance their education and training in more advanced countries. LMICs should increase their educational efforts, research capabilities, infrastructures, and living conditions to better serve their own populations and reduce their brain drain phenomenon.

## BRAIN DRAIN PHENOMENON: DEFINITIONS AND TRENDS

Brain drain is the migration of educated and skilled individuals from a less developed region or country to a more economically established one. It is defined in the Cambridge English dictionary as the loss of many highly skilled and educated people from one country to another.^1^ Brain drain is a critically important problem that unfortunately is not new. It starts with the ambition of the best and brightest, who, wishing to further their education, emigrate to accomplish this and do not return to their countries of origin for one or more reasons, including a lack of suitable opportunities and issues related to better income and quality of life. A significant number of talented individuals originating from low- and middle-income countries (LMICs) seek to advance their knowledge and skills in the various fields of science, technology, and medicine in industrialized high-income countries (HICs) and stay on to live and work because of greater financial reward or better opportunities for professional advancement. Many of these individuals excel in research and leadership and thus contribute to global advancements.

While WHO estimates the minimum number of health care workers required per 10,000 people to preserve the function of a health system to be 23,^2^ a large number of countries in the world still have health care worker shortages, and this is most pronounced in sub-Saharan Africa (SSA)^3^ from where there has been a persistent increase in physician migration.^3^ The United States, United Kingdom, Canada, and Australia are the four countries that have benefited most from the immigration of large numbers of physicians.^4^ The total number of international medical graduates in the United States is 208,733, 60.2% of which are from lower-income countries.^4^ India is the top source country and provides 40,838 physicians to the United States, which constitutes 4.9% of its physician workforce. India also provides the United Kingdom with 15,093 physicians, constituting 10.9% of its physician workforce.^4^ For the United States, the Philippines is the source of 17,873 physicians, Pakistan 9,667, China 6,687, Egypt 4,593, Mexico 4,578, South Korea 4,401, Lebanon (a country of only 4 million people) 2,556, Nigeria 2,392, and Colombia 2,363 physicians.^4^ Approximately 86% of African international medical graduates practicing in the United States originate from three countries: Nigeria, South Africa, and Ghana.^3,5^ Key reasons influencing doctors to emigrate are many, including poor salary, bad working conditions, lack of research funding, lack of advanced high-tech facilities, limited career development, unstable political climate, war, and poor academic training.^6^

In May 2019, the Trump administration proposed a merit-based immigration plan that would increase educational and skills requirements for people allowed to migrate to the United States.^7^ The consequential impact of such a merit-based US immigration program on health care delivery in LMICs should be addressed. Consideration of what impact this policy might have on countries from which these people emigrate should incite those countries to work on improving their educational and care delivery infrastructures to ensure that a needed significant proportion of their citizens who train abroad will return and contribute to the well-being of their populations and societies. As much as the new US immigration policies proposed by the Trump administration are designed to optimize the immediate benefit of immigration to the United States by profiling desirable immigrants for entry, the plan may have global implications with regard to education, medicine, engineering, information technology, industry, agriculture, and other fields. From a medical perspective, this approach also neglects the responsibility of the United States to contribute to improving global health and well-being.

## MEDICAL TRAINING AND GLOBAL ONCOLOGY CARE

The United States currently trains too few physicians to meet the country’s overall needs. By default, US health care relies on immigrant physicians to supplement the extension of its workforces into rural or underserved communities. The majority of skilled immigrants drawn to the United States by a merit-based policy would likely come from LMICs where few skilled employment options exist. The increasing flow of educated and skilled people from Africa, Asia, Eastern Europe, South America, and most LMICs would aggravate the brain drain phenomenon^3^ from which these countries already suffer significantly.

A common phrase in US health care these days is “Working at the top of your license.” Highly trained health care providers need to be able to productively use the entire scope of their knowledge and skill to feel personally fulfilled and for society to most effectively gain from the investment of resources in these individuals. When we bring physicians from LMICs to the United States and train them in accredited fellowship programs, we frequently raise their skillset to a level that cannot be fully realized in their countries of origin and create disincentives for them to return. People may stay in the United States rather than return to their countries of origin as a result of better living conditions and professional opportunities, as well as better access to medicines and technologies to treat diseases, such as cancer, compared with low salaries and poor infrastructure in their countries of origin. As long as there are major disparities in working conditions, research and practice infrastructure, and income, brain drain will continue. Individuals who decide to stay in the United States for professional and personal fulfillment are acting in a rational manner.

A merit-based immigration plan might worsen brain drain by facilitating the movement of individuals from LMICs who seek education and training in the United Sates and then stay once they have completed their training. LMIC policymakers already experience great challenges in maintaining, let alone improving, existing infrastructures and services.

## SOLUTIONS THROUGH INNOVATION IN MEDICAL EDUCATION, TRAINING, AND ADVANCING INFRASTRUCTURES

For many years, concerned physicians, professional societies, and universities have stressed the value of education, knowledge transfer, and capacity building in LMICs. ASCO, Breast Health Global Initiative, European Society for Medical Oncology, WHO, and many American and European academic institutions, among others, have developed educational programs for and in collaboration with physicians and medical teams from LMICs, emphasizing the importance of those individuals returning to their home countries to implement what they have learned. The goal has been to reduce health outcome disparities, advance technology, and promote infrastructure development in those countries that are at risk for brain drain.^8-11^

ASCO, Breast Health Global Initiative, and others have worked to organize on-site professional training in cooperation with health authorities and institutions in LMICs.^9,12^ LMICs will also have to prioritize the allocation of resources, optimize their health systems, and improve compensation and opportunities for educated and skilled people to retain them. Some institutions and countries—for example, Lebanon and Jordan—have succeeded in reducing brain drain in medicine by building medical centers and infrastructures that are accredited at high international standards, and by offering better compensation to accommodate their trainees sent overseas to attract their return.^13,14^

Short-term training exchanges, as opposed to formal enrollment in degree programs in HICs, can improve expertise within a country without increasing the risk of brain drain and can be optimized through long-term planning strategies. Policymakers and health authorities can consider short training periods overseas and support bringing international experts on missions and visits to train local physicians and nurses on site, working to increase collaboration with volunteer international societies. One successful example is the teaching module developed by the Society of Gynecologic Oncology of Canada for training gynecologists in LMICs to perform radical hysterectomy and pelvic lymphadenectomy.^16^

As much as it is important to continue knowledge exchanges and the training of physicians and surgeons from LMICs in the United States, Europe, and other industrialized nations, LMICs need to improve their own training facilities and programs as they upgrade their infrastructures and build their own institutions. For example, adopting the European Society for Medical Oncology/ASCO Global Curriculum for medical oncology training would allow LMICs to locally graduate better trained oncologists.^16,17^ In sub-Saharan Africa, the East Africa Center of Excellence in Oncology training program at the Uganda Cancer Institute is an example of a high-quality training effort that was funded, developed, and led within East Africa, with partners from HICs. The curriculum and faculty strive to meet high international standards, and current fellowships include pediatric oncology, gynecologic oncology, and adult hematology/oncology.^18^ Rwanda has developed a national cancer program by designing comprehensive integrated frameworks of care and building local human resource capacity through partnerships with institutions from HIC,^19^ and the East Africa Pediatric Oncology-Hematology Fellowship program was recently launched.^20^

One way for LMICs to counter medical oncology brain drain is to have a path for professionals to follow in their native countries that leads to exercising their acquired skillset in a creative and productive manner—a manner that allows them to maximize patient outcomes. LMICs can identify oncologic diseases with high incidences and prevalence and send their health care providers to HICs for targeted and shorter training specific to these cancers. When their providers return, LMICs can ensure adequate public health infrastructures and access to effective therapies where this new knowledge can be applied. Countries with high rates of cervical cancer might send out teams of gynecologic oncologists and radiation oncologists, and if lymphoma is the target, teams of hematopathologists and medical oncologists. Plans and policies from HIC and LMIC governments, US and European cancer centers, and private foundations might enable such programs.

Newly established health care centers in LMICs, particularly cancer care centers, have limited human and physical infrastructure and rely on developing a workforce through training in collaboration with established centers in more scientifically advanced institutions of industrialized nations. Education and training of health care professionals in LMICs can be done within their own countries or abroad. To address the global cancer burden, new approaches for incentivizing the oncology workforce to stay in or return to their countries are needed.

In India, the government launched a “no obligation to return to India” program and issues a “no obligation to return to India certificate” that is required for Indian doctors to settle in other countries.^21^ This step did not decrease the brain drain, but even seems to have worsened the doctor shortage.^21^ Instead, the Indian government should consider increasing postgraduation positions, improving infrastructure, and providing loans for setting up high-end infrastructure and developing a better medical curriculum.^21^ Other countries, such as Thailand and Ireland, have succeeded in reversing brain drain by offering generous research funding as well as services and assistance.^22^

According to the 2011 American Medical Association Physician Masterfile, the migration rate of sub-Saharan Africa–trained physicians increased from 2002 to 2011 for all sub-Saharan African countries except South Africa, which succeeded in decreasing the migration trend to the United States by 8%.^3^

## A CALL TO ACTION

It is hoped that this article serves as a call to action for countries who suffer from brain drain. We affirm that it is a fundamental human right that individuals have the opportunity to live, work, and raise future generations wherever their talents, labor, and ambitions lead them. In so doing they enrich the societies that welcome them. Societies should be able to compete for the best medical talent by creating attractive educational, research, and health care opportunities, whether through public government or private not-for-profit organizations; however, erecting barriers to the free flow of ideas and talent does a disservice to humanity. Ambitious individuals from LMICs need and should have the opportunities to advance their education and training in more advanced countries. When settlement and migration of highly trained individuals is facilitated and sought after via merit-based immigration policies in HICs, authorities in LMICs should recognize the need to increase their educational efforts and research capabilities and improve infrastructures and living conditions to better serve their own populations and reduce their brain drain phenomenon by attracting talent to return to their native countries. The United States and other countries, such as Canada, the United Kingdom, and Australia, will remain the leading destinations for LMICs medical graduates unless policies are implemented between HICs and LMICs to decrease the brain drain trend, thereby reducing the doctor shortage in the exporting countries.^3^ Another suggestion is that the HICs might have to consider reimbursing LMICs for the cost of training the health care professionals they import.^22^

This problem has long existed, and although potentially exacerbated by merit-based immigration policies in industrialized nations, the focus should be on a call to action on concepts that address the root causes of brain drain, reduce disparities, and ensure global scientific exchanges and human development.
